# Lobular breast cancer and prognosis: what should we tell patients?

**DOI:** 10.3389/fonc.2026.1877790

**Published:** 2026-09-04

**Authors:** Tivya Kulasegaran, Kate Beecher, Pamela Kinnon, Sunil R. Lakhani, Peter T. Simpson, Amy E. McCart Reed

**Affiliations:** 1 Fraser Institute, Faculty of Health, Medicine and Behavioural Science, The University of Queensland, Brisbane, QLD, Australia; 2 Cancer Care Services, Metro North Hospital and Health Service, Brisbane, QLD, Australia; 3 Lobular Breast Cancer Alliance Australia and New Zealand, Brisbane, QLD, Australia; 4 Pathology Queensland, Royal Brisbane and Women’s Hospital, Brisbane, QLD, Australia; 5 School of Biomedical Sciences, Faculty of Health, Medicine and Behavioural ScienceThe University of Queensland, Brisbane, QLD, Australia

**Keywords:** breast cancer, ILC, invasive lobular carcinoma, lobular, outcome, survival, prognosis

## Abstract

**Background:**

Invasive lobular carcinoma (ILC) is the second most common subtype of breast cancer. It accounts for up to 15% of all breast cancer diagnoses, and there is an increasing population of people living with a diagnosis of ‘lobular’. ILC has historically been understudied and is underrepresented in clinical trials. Presenting with pathology features typical of a good prognosis (oestrogen receptor-positive, HER2-negative, grade 2), a diagnosis of ILC can be framed as one with a ‘good outcome’. But whether the data support this over time remains to be seen.

**Methods:**

We present a comprehensive narrative review to ascertain whether ILC has a better outcome than invasive carcinoma of no special type (IC-NST).

**Results:**

A number of the research studies analysed showed that ILC can have a better outcome at 5 years and still have a poorer outcome over time, whilst others showed that IC-NST has a poor outcome early, which is maintained over time.

**Conclusions:**

We discuss limitations and confounders and, critically, give voice to the people living with a lobular breast cancer diagnosis.

## Introduction

Breast cancer (BC) encompasses a wide range of tumour subtypes that can be classified based on the histopathological features, and molecular biomarkers. The most common type, which accounts for 80% of all BCs, is invasive carcinoma of no special type (IC-NST; formerly invasive ductal carcinoma, IDC). Invasive lobular carcinoma (ILC) is the second most common subtype and constitutes 15% of all invasive breast malignancies (1).

Clinically, ILC differs from IC-NST. ILC tends to be diagnosed later in life and often presents with a larger tumour size (2). It is more likely to be multifocal at the time of diagnosis and can exhibit a distinct metastatic pattern, with an increased likelihood of dissemination to the peritoneum, gynaecological organs, meninges, and the ocular orbit. Despite the unique biology of ILC, its therapeutic management is the same as that for IC-NST.

ILC is frequently described as the ‘good prognosis’ BC, typically oestrogen (ER) and progesterone receptor (PR)-positive, HER2-negative, and grade 2, with a low proliferative index. The 5-year survival outcomes are often favourable, and long-term survivorship is common. However, there is also increasing contradictory evidence, with numerous studies reporting a high rate of late relapse. Conflicting registry data to this effect have also created uncertainty. For individuals living with ILC, this discordance between early reassurance and later risk can generate confusion and anxiety.

That we can discuss long-term survival at all for lobular BC is fortuitous. Survival does not need to be measured in months, like for a harrowing pancreatic or glioblastoma diagnosis; however, neither it is a diagnosis to be taken lightly. Anecdotes of clinical teams celebrating a diagnosis of the ‘lucky breast cancer’ abound and justifiably rankle those living with a diagnosis.

At 15% of all BC diagnoses, we can expect an incidence of at least 48,000 new ILC cases in 2026 in the USA alone (3). In fact, large-scale meta-analyses have shown that ILC is increasing in incidence (2). We now see an emergence of effort to understand ILC biology, and the increasing need for education is evidenced by the ‘Lobular session’ (Special Session 2: Lobular Breast Cancer Updates) at San Antonio Breast Cancer Symposium 2025 and the coming together of focussed consortia (4–6). Over the years, there have been numerous conflicting reports about the overall survival (dis)advantage of ILC. Herein, we curate the available data. Understanding its true long-term prognosis is therefore not a niche question, but one with substantial clinical and survivorship implications.

## Methods

### Narrative review approach

**Figure 1:**
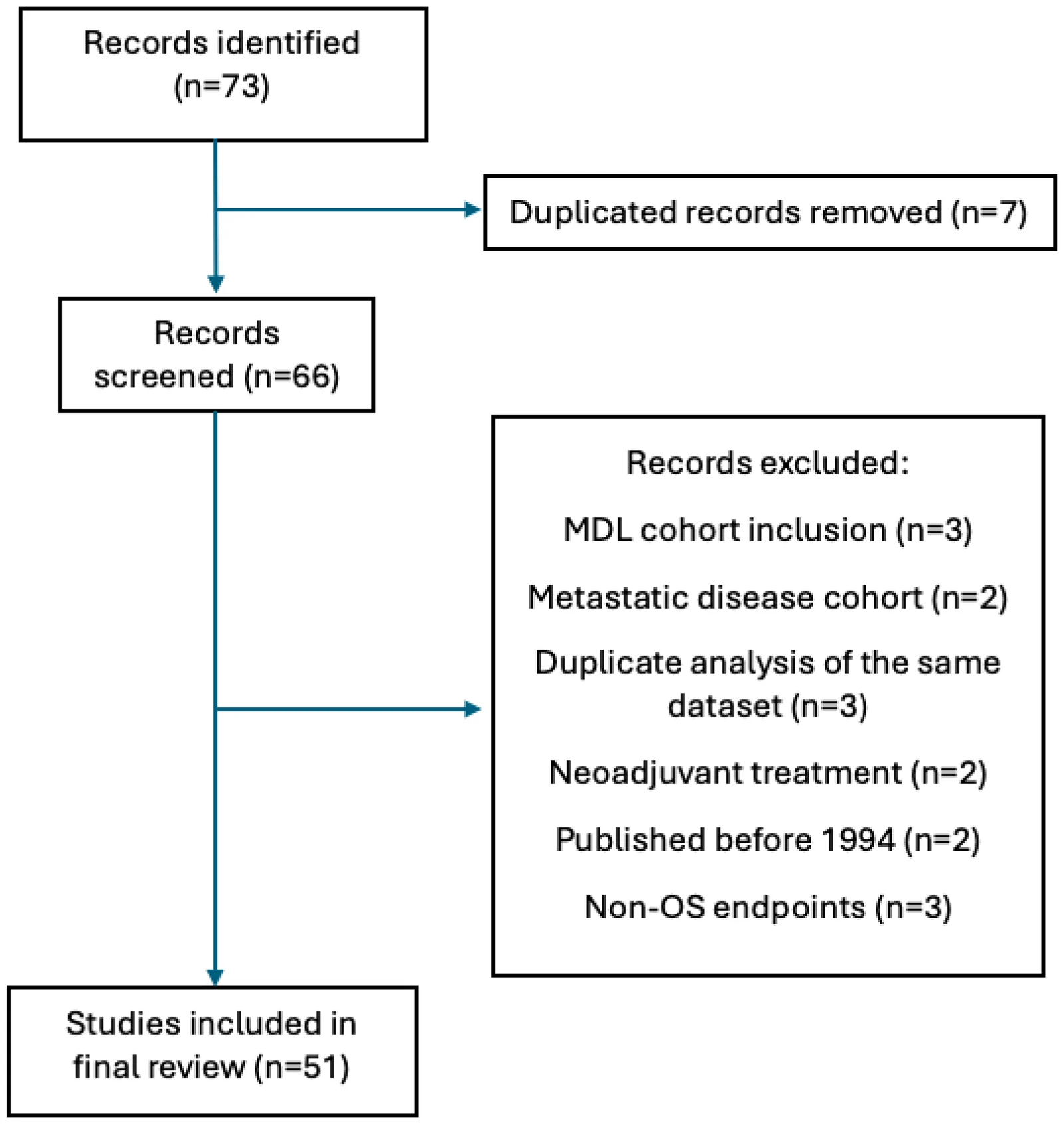
Modified preferred rporting items for systematic reviews and meta-analyses (PRISMA) schema detailing the study selection. MDL, mixed ductal lobular; OS, overall survival.

Using PubMed with the search terms ‘lobular breast cancer’ and ‘invasive lobular carcinoma’, we retrieved articles and examined their relevance for outcome data (not just incidence). **Figure 1** shows a modified Preferred Reporting Items for Systematic Reviews and Meta-Analyses (PRISMA) flowchart. We restricted inclusion to a relatively contemporary time period (12–62), excluding (7) and (8), and required a minimum median follow-up of 48 months. Papers with a second analysis of an already published datasets were omitted (9–11).

The Surveillance, Epidemiology and End Results Program (SEER) data analysis in (2) did not specify inclusions or patient numbers and thus was excluded. However, we did include nine SEER analyses with differing inclusion criteria and matching parameters. We also excluded neoadjuvant, metastatic, and those studies comparing ILC to mixed ductal–lobular (MDL) carcinoma. Despite these exclusions, a meta-analysis was still precluded by the lack of homogeneity in data collection. Outcome was variously measured as overall survival (OS), disease-free survival (DFS), recurrence-free survival (RFS), excess mortality rate (EMR), breast cancer-specific survival (BCSS), and locoregional recurrence (LRR). Equally, the follow-up periods differed, and the inclusion criteria for individual studies varied. Therefore, the standard of care therapy may differ in each cohort (e.g., inconsistent tumour stage). Nevertheless, given that ILC is broadly homogenous for clinical features (ER-positive, HER2-negative, and grade 2) and is managed according to international guidelines, these are taken as acceptable limitations for a qualitative comparison.

Following article review, we noted whether the data were from a trial or registry, the cohort size, and the period of study. We then recorded survival data, the length of follow-up, and whether any analyses were statistically significant and, if so, which group had better outcome. Studies were categorised into: i) ‘Yes’ = ILC showed better survival than IC-NST throughout the reported follow-up; ii) ‘No’ = ILC showed equivalent or worse survival; and iii) ‘Yes then No’ = ILC showed an early survival advantage (typically <5 years) that reversed to a notably poorer outcome in later follow-up. As such, studies with shorter follow-up could only be classed as ‘Yes’ or ‘No’. Where formal time-stratified hazard ratios (HRs) were unavailable, categorisation was based on explicit author descriptions of time-dependent reversal of survival advantage.

### Literature synthesis

A total of 51 informative studies, covering 41 datasets, are summarised in **Supplementary Table 1**. The median number of cases in each study was 451 (range = 30–39,859). Of the 51 included studies, 46 are registry studies, with four clinical trials and one study unclear. Single-institution studies comprised 57% (29/51) of the datasets. Typically, cases were coded as ILC or IC-NST based on the International Classification of Diseases for Oncology (ICD-O) codes 8520 and 8500, respectively, with a range of exclusions detailed where available.

**Figure 2:**
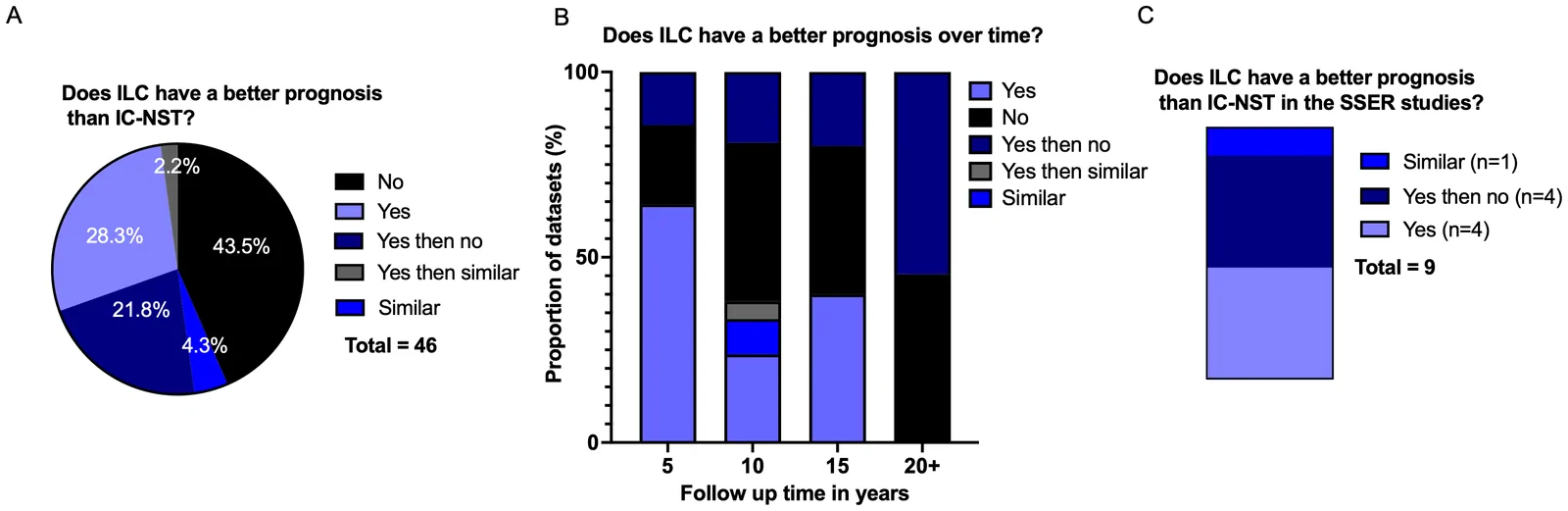
Invasive lobular carcinoma (ILC) prognosis can be worse in the longer term than invasive carcinoma of no special type (IC-NST). **(A)** Total breakdown of the outcomes in 46 registry datasets by percentage. **(B)** Breakdown of the prognostic outcomes stratified by the length of follow-up time in years. **(C)** Surveillance, Epidemiology and End Results Program (SEER) studies (*n* = 9) despite having overlapping cohorts, stratified by prognostic outcomes. Categories defined as: ‘Yes’ = ILC better throughout follow-up; ‘No’ = ILC not better; ‘Yes then No’ = early advantage lost over time. Note that studies with shorter follow-up (<5 years) can only be classed as ‘Yes’ or ‘No’.

In a crude assessment of OS, shown in **Figure 2A**, almost two-thirds of the registry studies demonstrated that ILC does not have a better OS than IC-NST (43.5% ‘No’ and 21.8% ‘Yes then No’). In studies matching for ER status, two out of six studies support ILC to have a poorer overall outcome, and a further two studies indicate that ILC has a worse outcome over time (‘Yes then No’). The data supporting ILC as having a better prognosis is complicated over the longer term. There were 10 studies reporting an early survival advantage for ILC (<5 years), with a change to a poorer prognosis overtime (‘Yes then No’). **Figure 2B** shows the data over follow-up periods (binned into 5-year groups). Of the eleven studies with more than 20-year follow-up data, 70% reached statistical significance. Five studies showed ILC with a poorer outcome and six showed an early ILC advantage that converted to poorer outcomes. At 15 years, two out of three studies showed an advantage for ILC, one showed no difference, and three showed ILC to have a poorer outcome. At 10 years, five out of 16 studies showed that ILC does better, two showed no difference, five showed a poorer outcome for ILC, and seven showed an early advantage that converts to a poorer outcome. There were 20 studies reporting on a 5-year window, with 17 showing a survival advantage for ILC.

Considering only relatively contemporary studies involving patients diagnosed in the year 2000 or after, there were 11 registry studies that had maximum reported follow-ups of 10 years. Of these 11 studies, seven reported a poorer outcome for ILC, with three out of seven reaching statistical significance and another two showing an early survival advantage followed by a decline to a poorer prognosis. There were three studies that presented data showing ILC to have a better OS, with two out of three reaching statistical significance.

### Methodological approaches

Across the included studies, a range of approaches to refine and analyse an eligible cohort analyse were encountered. There were nine studies that analysed the SEER dataset, each applying slightly differing inclusion criteria and producing conflicting findings: in two studies, the survival between IC-NST and ILC was similar (12, 13), three studies reported better survival for ILC over IC-NST (14–16), and the remaining four studies demonstrated a decline in survival advantage for ILC over time (17–20) (**Figure 2C**). A propensity score matching (PSM) approach to account for confounding factors was applied by four out of nine of these SEER analyses. The large Yang et al. study of the SEER data (13) (*n* = 30,190 ILC and *n* = 288,216 IC-NST) reported a similar OS for ILC and IC-NST; however, after PSM for ER/PR-negative, N2/N3 nodal stage, and TNM stage III, ILC had a poorer outcome. This was not the case for ER/PR-positive matching. Giaquinto et al. (2) reviewed data from SEER and the Center for Disease Control and Prevention’s National Program of Cancer Registries and determined that ILC had a better outcome at 7 years, equal at 10 years, and poorer for ILC over longer follow-up, with higher rates of regional and distant-stage disease.

A further four studies applied PSM in their analyses. The Han study (21) showed that ILC had worse OS, and similar results were obtained both with and without PSM for age, size, LN status, ER/PR/HER2, and Ki67. Magnoni et al. (22) analysed data from a clinical trial testing abemaciclib in high-risk BC patients, which, after matching, included 361 (23.7%) out of a total of 1,524 patients with ILC. This showed improved OS for ILC in the setting.

A worse outcome after chemotherapy was noted for ER/PR-negative ILC in the Yang study (13), as well as for ER/PR-positive ILC (23). A poorer outcome for ILC was also reported when lymph nodes are positive (18, 24). The Yoon et al. study (20) analysed three separate datasets including SEER and focussed on premenopausal women. In each dataset, the early BCSS (<5 years) showed a survival advantage for ILC. However, with time, this advantage was lost, with ILC doing more poorly.

As much as we can match grade and stage, amongst other clinical variables, it bears repeating that ILC is typically diagnosed larger and later (2), and it is important that, when matching, we focus on the most representative groups clinically.

### Clinical and lived experience implications

That the ILC outcome data are inconsistent is not merely a frustration for scientists and clinicians. There is also the impact on consumers to be considered. Early reassurance from clinicians and positive 5-year prognosis should not replace disclosure of the clinically relevant future risk. Indeed, Ms. Pamela Kinnon described her diagnosis of ILC as being told she had the ‘lucky cancer’. Consumers noted that discussion of the biological behaviour of ILC is sometimes avoided in clinical appointments or is summarised with the anthropomorphist description as being ‘sneaky’ cancers. Regardless of whether this is to minimise patient anxiety, it is not appropriate. Consumer advocates also reported confusion around prognosis when being told their type of cancer is ‘the good one’, but then reading that there is still a risk of metastasis and that the detection methods are not completely reliable.

Survival statistics are not abstract measures: they inform counselling at diagnosis, surveillance strategies, and survivorship planning. For ILC, early reassurance based on 5-year survival may not fully capture the long-term recurrence risk. More than half of the studies included here demonstrated a late decline in survival relative to IC-NST, particularly beyond 10 years. From a lived experience perspective, uncertainty regarding long-term risk can be distressing, particularly when expectations are set early around a favourable prognosis. A clearer understanding of time-dependent outcomes may improve patient understanding, potentially influencing extended endocrine therapy decisions and surveillance considerations.

Importantly, consumer advocacy has been central in bringing this clinical need to light. In sum, information should be communicated accurately and proportionately, with survivorship support and psychosocial care offered according to individual need.

### Limitations

That we are precluded from a thorough meta-analysis is disappointing, but allows us to consider the key frameworks for establishing larger studies moving forward. PSM hints at a similar outcome for ILC and ER-positive IC-NST, which needs confirmation. Moreover, we need to ensure representation for the common ILC presentations in matching criteria. Clear annotation of any ILC histological variants remains a key component of any ILC registry analysis, and we need to be sure we are defining cohorts with care and clarity (25, 26). In October 2016, the American Joint Committee on Cancer (AJCC) released the eighth edition of the tumour staging manual, updating to include biomarkers and molecular tests (reviewed in (27)). As a consequence, the staging for a subset of tumours has changed depending on whether the seventh or eighth edition is used. Thus, tumours diagnosed and staged from 2017 onwards must be compared with care to those staged using the seventh or earlier editions. Finally, using standard measures of survival such as OS or BC-specific survival can help to bolster and leverage the existing literature: detailed definitions for recorded measures are essential. Taken together, a single-institution study with minimum 15 years of follow-up, contemporary treatment modalities, and detailed annotation is needed for a clearer picture of the impact of lobular histology on survival.

## Key conclusions

The 5-year survival in ILC is frequently favourable, but does not reliably predict long-term outcomes. A late change in the prognosis of ILC has been reported by a number of studies; however, methodological heterogeneity, particularly short follow-up periods and registry design, may obscure time-dependent risks. As such, we propose that ILC should not be uniformly framed as a ‘favourable’ subtype without acknowledging long-term considerations.

## Supplementary material

Supplementary Table 1 Summary of ILC survival data.
